# Motives and Barriers to Exercise Training during Hospitalization in Patients with Type 2 Diabetes: A Cross-Sectional Study

**DOI:** 10.3390/ijerph19031035

**Published:** 2022-01-18

**Authors:** Stig Molsted, Lasse Kusk, Søren Mingon Esbensen, Thomas Martin Mohr, Malene Bang Vind, Camilla Hess, Thomas Bandholm, Morten Tange Kristensen, Cornelie Mølsted Flege, Peter Lommer Kristensen

**Affiliations:** 1 Department of Clinical Research, Nordsjællands Hospital, 3400 Hillerød, Denmark; lasfarr@gmail.com (L.K.); soren.mingon@gmail.com (S.M.E.); barbelltreatment@gmail.com (T.M.M.); 2 Department of Clinical Medicine, Faculty of Health and Medical Sciences, University of Copenhagen, 2200 Copenhagen, Denmark; Thomas.Quaade.Bandholm@regionh.dk (T.B.); morten.tange.kristensen@regionh.dk (M.T.K.); Peter.Lommer.Kristensen.01@regionh.dk (P.L.K.); 3 Department of Endocrinology and Nephrology, Nordsjællands Hospital, 3400 Hillerød, Denmark; malene.bang.vind@regionh.dk (M.B.V.); camilla.engelstoft.hess@regionh.dk (C.H.); 4 Department of Clinical Research, Department of Physical and Occupational Therapy, Department of Orthopedic Surgery, Copenhagen University Hospital, Amager and Hvidovre, 2650 Copenhagen, Denmark; 5 Department of Physical and Occupational Therapy, Copenhagen University Hospital, Bispebjerg-Frederiksberg, 2400 Copenhagen, Denmark; 6 Department of Food Science and Technology, Faculty of Science, University of Copenhagen, 1870 Frederiksberg, Denmark; cornelieflege@gmail.com

**Keywords:** type 2 diabetes, admission, exercise training, hospitalization, physical function, physical activity, motives, barriers

## Abstract

Background: Exercise training during hospitalization may prevent loss of physical function and hyperglycemia in patients with type 2 diabetes. The aim of this study was to assess motives and barriers to exercise training in hospitalized patients with type 2 diabetes. Methods: Data were collected using a questionnaire about motives and barriers to exercise training during hospitalization. Additional data for clinical characteristics of the participants were collected from patient records. Results: 79 patients participated (mean ± SD age 72 ± 12 years; 42% women), of whom 25% had a low level of education and 46% lived alone. The median (IQR) length of the stay was 6 (4–10) days. A total of 67% of the participants wished to be more physically active. Walking as exercise was preferred by 51%. The most frequently reported barriers to exercise training were bodily pain (48%) and dizziness (42%). Low vs. high level of education, and living alone vs. being married/living with a partner were associated with reduced odds of a wish to be more physically active, odds ratio (OR) 0.15 [95% CI 0.03; 0.76], *p* = 0.022, and 0.21 [0.05; 0.82], *p* = 0.025, respectively. Conclusion: Two out of three hospitalized patients with type 2 diabetes wished to be more physically active during admission. Bodily pain was a barrier to exercise training and needs attention in training programs. As a low level of education was associated with reduced odds of a wish to be more active, a strategy to include all patients in training programs which considers social inequality is needed.

## 1. Introduction

Hospitalization is often associated with sedentary behavior [1]. A very low level of physical activity may, even in the short term, affect physical function and the course of the admission negatively. When sedentary behavior happens in concert with type 2 diabetes mellitus (T2DM) and old age, the detrimental effects may be even greater [2]. Thus, early initiation of physical activity to elderly inpatients with T2DM has been recommended as key action to prevent loss of physical function as well as falls [3].

The negative effect of sedentary behavior, T2DM and old age on physical function is the result of a loss of muscle mass [4,5,6]. In addition, an acute illness may together with sedentary behavior also lead to hyperglycaemia [7]. The impaired glycaemic control during hospitalization is usually treated with short acting insulin many times per day, which represents a time-consuming task for nurses and is most likely associated with a risk of high glycaemic variability and hypoglycaemia.

Whilst physical activity and exercise training may prevent loss of muscle mass and physical function, it may also act as a treatment of hyperglycaemia. Exercise training is a corner stone in the treatment of T2DM and has significant effect on hyperglycaemia [8,9,10]. Thus, exercise training during admission may not only prevent loss of physical function, but it may also prevent hyperglycaemia.

Many countries are undergoing demographic changes, with an increasing number of elderly citizens. As higher age is associated with an elevated risk of T2DM and other chronic diseases, the pressure on the healthcare systems is increasing. New initiatives are therefore needed to be tested to decrease the length of hospitalizations, and to prevent re-admissions after a hospitalization. A more intensive and structured exercise training during hospitalization may be a very important intervention to improve these abovementioned outcomes.

A previous study found that hospitalized patients with T2DM walked less than half of the number of steps compared to non-hospitalized patients with T2DM [11]. Structured exercise training during admissions could potentially increase the level of physical activity. However, several factors including patients’ motivation for exercise during hospitalization could be barriers and more knowledge of those issues are needed to design new interventions and studies to increase physical activity levels during hospitalization. Thus, the aim of the present study was to assess motives and barriers to exercise training in admitted patients with T2DM.

## 2. Materials and Methods

This cross-sectional study was conducted from September 2020 to May 2021 at The Department of Endocrinology and Nephrology, and The Department of Cardiology, Nordsjællands Hospital, Denmark. The study was paused from the 26th of November 2020 to the 12th of January 2021 due to the COVID-19 pandemic. The inclusion criteria were age ≥18 years; T2DM; and understanding and speaking Danish. The exclusion criteria were any surgery during admission; limb amputation; any contraindication to standing and walking; blindness; and severe psychiatric disorder.

Potential participants were found on weekdays during morning meetings with departmental staff. After screening of inclusion and exclusion criteria, participants were approached and asked about their interest in participating. Data were collected using a structured questionnaire that was completed by the interviewers via patient interviews performed by LK, SME, TMM, and MBV. The interviews lasted ~10 min and were performed on the second day of admission or as soon as possible thereafter.

### 2.1. Outcome Variables

The questionnaire included questions about patients’ motives towards exercise training, wishes, physical function, etc. The questionnaire was not tested for validity and reliability prior to the study. The questions were constructed according to what the researchers intended to collect data on.

The questionnaire comprised the following questions and response options:

1. Desires regarding a higher level of physical activity during admission (yes/no/do not know);
2. Motivation towards exercise training during admission (numeric rating scale 0–10);
3. Motives towards exercise training during admission (open question);
4. Desires regarding exercise during admission (walk; exercise in the hospital room; exercise in a fitness room);
5. Desires regarding daily exercise duration during admission (min);
6. Desires regarding exercise training setup during admission (individual training; supervised training; group training).

Question 2 on motivation was a supplement to question 1 and was added to investigate the level of motivation. Question 4 was included to focus on the exercise training options in the hospital department.

Barriers were also covered by previously presented questions from Moreno NA and colleagues [12], and the results from the present study were compared with data for older admitted patients (n = 68, age > 60 years, different diagnoses, hospitalized at a department for respiratory and clinical medicine, Brazil, in that study [12].

### 2.2. Other Variables Collected Using the Questionnaire

- Physical mobility during admission (need of support and aid);
- Dizziness (yes, very bothered; yes, bothered a little; no dizziness);
- Socioeconomic status (three levels of education) [13];
- Marital status (married/living with a partner; living alone) [13].

Socioeconomic and marital status were assessed using questions from the Danish Health and Morbidity Survey [13].

### 2.3. Other Variables Collected in the Patient Records

Data on age, sex, reason for the admission, body mass index, length of hospitalization, blood pressure (at time of to the interview), and glycaemic control based on HbA1c were collected.

### 2.4. Ethics

The participants gave their written informed consent and the study was performed in accordance with The Helsinki Declaration, rules of the local ethical committee and the Data Protection Agency (P-2020-734).

### 2.5. Statistical Analyses

The number of included patients was based on a pragmatic approach with a limited inclusion period, where students were working on the study. Descriptive data are presented as number (percentage), mean ± standard deviation (SD), or median (interquartile range (IQR)), depending on the type and distribution of data. The barriers to physical activity of the included patients were compared with data for a previous sample of older admitted patients presented by Moreno and colleagues [12] using a Chi^2^ test. The internal consistency reliability of the present data from the method of Moreno was assessed using Cronbach’s α. The differences in reported barriers and level of motivation for exercise training in subgroups of patients were tested using a Chi^2^ test and Student’s unpaired t-test. The associations between the exposure variables (age, sex, educational level (low, medium or high), marital status, and HbA1c) and the outcome variable ‘wish to be more physical active during admission’ were analyzed using a multiple logistic regression model. In the regression model, patients with ‘other’ educational level (n = 2) were removed. The results of the regression model are presented as odds ratio (OR) [95% confidence interval (CI)]. All statistical tests were considered significant (2-tailed) when *p* < 0.05. The statistical analyses were performed using the IBM SPSS 25 program.

## 3. Results

The patients’ characteristics are presented in Table 1. A total of 160 patients with diabetes were screened to be recruited, 14 patients did not wish to participate, 26 patients were discharged before inclusion, and 42 patients were excluded (amputation n = 7; dementia/cognitive dysfunction n = 14; type 1 diabetes mellitus n = 6; surgery during admission n = 6; psychiatric disease n = 4; did not understand Danish n = 4; and tetraplegia n = 1); thus, 79 patients were included in the study.

### 3.1. Motives and Barriers to Physical Activity

The included patients were 72 ± 12 years old, 42% were women, and they were hospitalized for 6 (4–10) days in total.

When the barriers were assessed by the method of Moreno and colleagues [12], the internal consistency reliability of that part of the questionnaire was Cronbach’s α = 0.619. The most frequently reported barriers to physical activity during admission were ‘Pain in any part of the body’, ‘Dizziness’ and ‘Dyspnea’ (Table 2). ‘Lack of companion encouragement’ and ‘Fear of losing venous access’ were the least frequently reported barriers. Patients with T2DM from the present study reported more frequently that bodily pain, dizziness, and fear of falling were barriers to physical activity compared to data reported by the cohort of older admitted patients (without diabetes) with different underlying diseases (Table 2).

A total of 67% of the patients wished to be more physically active during admission (Table 3). The most frequently reported preferred exercises were walking, mixed exercises on their hospital ward and aerobic training, whereas mixed exercises in groups were least frequently reported. The most frequent motives for exercise training were to increase strength, quality of life and general health. Training alone with supervision from a physiotherapist was the preferred exercise training program setup (Table 3).

The motivation for exercise training assessed using the numeric rating scale was 6.79 ± 2.59 in patients who wished to be more physically active vs. 3.30 ± 2.66 (*p* < 0.001) in those who reported that they did not wish to be more physically active. While more patients who did not wish to be more physically active reported ‘Fear of losing venous access’ compared to those who wished to be more physically active (n = 4 (20%) vs. n = 1 (2%), *p* = 0.006), there were no differences between the two groups’ reports of other barriers (Table 2).

### 3.2. Associations between Different Variables and a Wish to Become More Physical Active

Having a low level of education (compared to a high level) was associated with reduced odds of a wish to become more physically active during hospitalization; likewise, living without a partner was associated with reduced odds off the same wish (Table 4). Age, sex and glycaemic control were not associated with a wish to become more physically active.

## 4. Discussion

This study found that two thirds of the admitted patients with T2DM wished to perform physical exercise training during hospitalization. Low educational level and living without a partner were associated with a reduced wish to exercise.

A positive result in this study was that most hospitalized patients with T2DM wanted to take part in exercise during hospitalization as this may prevent—at least to some extent—a loss of physical function before discharge and return to daily living. A recent study by Martínez-Velilla and colleagues found that supervised resistance, balance and walking training were safe and improved physical function in old patients with T2DM [14]. Our data add to this finding by identifying different barriers, among them pain, dizziness, and fear of falling, that may prevent patients from exercising during hospitalization. Addressing these issues on an individual basis will probably increase participation in future hospital-initiated exercise interventions. Fazio and colleagues reported in a review the magnitude of inactivity during adults’ hospitalization, and that a more intensive intervention than mobilization to a sitting position in a chair could be improved with more active exercises [1]. The patients’ positive attitudes to exercise training in this study support interventions with higher levels of physical activity than regular mobilization in the ward.

In the present study, the patients’ median length of stay in total was six days, where the median stay of patients was one day in the emergency department and five days in the ward. Five days with physical inactivity (and illness) may be associated with a significant risk of physical function loss, however, it also provides the opportunity for a preventive exercise training intervention. As different exercise modalities were preferred by the participants, departments should offer different kinds of exercise. Walking was suggested by most participants and this could be performed on a treadmill in the ward or just using the space in the ward. Regardless of exercise modalities, supervision by staff should be considered to ensure intensity, adherence, and motivation, and to prevent injuries. However, self-managed interventions should also be considered as the resources to new interventions may be difficult to provide. In addition, the promotion of mobility can be difficult in terms of responsibility and should be planned [15].

A low educational level was associated with reduced wish to exercise training. Thus, approaches should be considered to avoid social inequality in an exercise training intervention. It may be important to make simple information available to the patients about the importance of exercise training during an admission. Again, if it is feasible, the supervision of the exercises may be crucial to include as many patients as possible, especially those with a low socioeconomic status. Whilst a patient with a higher socioeconomic status may be motivated to exercise and has the skills to perform it, a patient with a low socioeconomic status may need more information about the issue to be motivated, and may indeed need supervision to perform it.

Bodily pain was reported as a barrier to exercise training in this study. Previous studies have suggested that musculoskeletal pain is frequent in patients with T2DM [16] and that it is associated with reduced physical activity levels [17]. However, exercise training may have the potential to decrease musculoskeletal pain in patients with T2DM [18], which is an important message to patients (and exercise supervisors). Supervision or instruction of the exercises and adequate use of painkillers may be important to ensure that the patients feel safe during the intervention and to suggest exercises that can be performed with reduced pain.

Whilst exercise training during admissions may prevent loss of physical function in patients with T2DM [12], it may also have another positive effect. Acute illness and physical inactivity may lead to hyperglycaemia, which is associated with poor outcomes [19,20]. Hyperglycemia during admissions in patients with T2DM is often treated with insulin, which is considered a ‘high-risk’ medication due to the risk of hypoglycaemia. A structured daily exercise training program may counteract hyperglycemia and reduce the need for insulin treatment.

This study is limited using a questionnaire that was not tested for validity and reliability in all questions. The study may also have potential selection bias as it was not possible to approach all hospitalized patients with T2DM on all days. Furthermore, the results cannot be extrapolated to hospitalized patients suffering for other diseases, for example stroke and fractures. The strength of the study was the pragmatic approach with few exclusion criteria which make the results more generalizable.

The results have clinical implications and address important barriers to exercise training to admitted patients with T2DM. Even though it has been recommended that exercise training is initialized early in clinical practice [3], more data on how to implement realistic interventions are needed. In a future study, training interventions should be tested to investigate adherence and effects on physical function and other outcomes during and after hospitalization. Exercise training interventions should also be tested together with the use of a physical activity tracker that may improve the effect on physical activity. as suggested by Pezzino and colleagues [11]. A previous study by Pupier and colleagues found significant but not very large differences between the physical activity level in patients with T2DM when they were hospitalized and at home [21]. However, in the study by Pupier and colleagues, the patients were only included if they did not have peripheral neuropathy, arterial diseases or articular pathology, and the sample may represent more healthy patients than those who were included in the present study. Patients’ goal setting and feedback methods have also been suggested to support behavioral changes and improve outcomes [22]. As exercise training may reduce the blood glucose in patients with T2DM, there is a risk of hypoglycemia, especially if the patients are treated with insulin.

## 5. Conclusions

In conclusion, two out of three admitted patients with T2DM wished to be more physically active during their admission and most preferred walking as an exercise modality. As bodily pain was one of several barriers to exercise training, information of safety and motivational supervision of the exercises may be important. A low level of education was associated with a reduced wish to exercise and inclusion of those with a low level of education should be emphasized to avoid social inequality when exercise training interventions are tested.

## Figures and Tables

**Table 1:** Characteristics of the participants.

Variables	n = 79
Age (year)	72 ± 12
Sex (women),(n)	33 (42%)
HbA_1c_ (mmol/mol)	67 ± 27
Educational level (n)	
Low	20 (25%)
Medium	29 (37%)
High	28 (35%)
Other	2 (3%)
Marital status (n)	
Married/living with partner	43 (54%)
Living alone	36 (46%)
Glucose lowering treatment before admission (n)	
None	11 (14%)
Oral, insulin, liraglutide or other treatment	89 (86%)
Body mass index (kg/m^2^) ^a^	28.9 ± 6.7
Blood pressure, systolic/diastolic (mmHg)	131 ± 27/70 ± 12
Admission days at testing time (n)	2 (1–4)
Admission days before endocrinology department (n)	1 (0–1)
Admission days at endocrinology department (n)	5 (3–7)
Admission days in total at the hospital (n)	6 (4–10)
Physical function (n)	
Can move from laying to sitting position without assistance	65 (82%)
Can move from sitting to standing position without assistance	65 (82%)
Can stand without support	59 (75%)
Can walk without support	48 (61%)
Needed support to walk during admission(n)	
From walker (or similar with wheels)	30 (38%)
From cane	3 (4%)
Reason to admission (n)	
Dysregulated diabetes	14 (18%)
Heart disease	14 (18%)
Infection	8 (10%)
Respiratory symptoms	8 (10%)
Kidney disease	8 (10%)
Gastrointestinal symptoms	6 (8%)
Other	21 (27%)

Data are presented as mean ± SD, median (IQR) or n (%). ^a^ missing n = 6.

**Table 2:** Self-reported barriers to physical activity during admission in patients with type 2 diabetes and older patients with other diseases.

Barriers to Physical Activity during Admission	The Present Study (n = 79)	Older Admitted Patients (n = 58) [12]	*p*
Pain in any part of the body	38 (48%)	18 (31%)	0.045
Dizziness	33 (42%)	11 (19%)	0.005
Dyspnea	32 (41%)	28 (48%)	0.365
Fear of falling	30 (38%)	5 (9%)	<0.001
Lack of professional help	28 (35%)	24 (41%)	0.479
Lack of equipment	24 (30%)	9 (16%)	0.044
Use of intravenous medication	23 (29%)	7 (12%)	0.017
Unwillingness to move	22 (28%)	11 (19%)	0.230
Lack of space	19 (24%)	44 (76%)	<0.001
Fear of infections	16 (20%)	29 (50%)	<0.001
Fear of missing a doctor’s visit	14 (18%)	1 (2%)	0.003
Lack of companion encouragement	7 (9%)	26 (66%)	<0.001
Fear of losing venous access	5 (6%)	13 (22%)	0.006

Data are presented as n (%).

**Table 3:** Motives to be physical active during admission.

Wished to be more physical active during admission	n (%)
Yes	53 (67%)
No	20 (25%)
Don’t know	6 (8%)
Motivation for exercise training (0–10)	5 (4–8)
Preferred light exercise training	
Walking	40 (51%)
Mixed exercises on hospital ward	26 (33%)
Aerobic training (ergometers)	22 (28%)
Stair climbing	9 (11%)
Mixed exercises in groups	5 (6%)
Motives to be more physical active during admission	
More strength, better QOL, better general health	48 (61%)
Medical reasons	9 (11%)
Nonspecific	26 (33%)
Wishes to organization of exercise training	
Training on their own	25 (32%)
Training alone with supervision from physiotherapist	39 (49%)
Training in groups with supervision from physiotherapist	29 (37%)
Preferred duration of daily exercise training during admission (min)	30 (15–30)

Data are presented as n (%).

**Table 4:** Multiple logistic regression model with the associations between the independent variables: socioeconomic status, marital status, HbA1c, age, sex and the dependent variable ‘wish to be more physically active during admission’.

Independent Variables	Wish to Be More Physically Active	
	OR [95% CI]	*p*
Age (years)	0.97 [0.92; 1.03]	0.327
Sex (female)	2.67 [0.65; 11.04]	0.174
Socioeconomic status		
High	Reference	
Medium	2.38 [0.51; 11.20]	0.273
Low	0.15 [0.03; 0.76]	0.022
Marital status (living alone)	0.21 [0.05; 0.82]	0.025
HbA1c (mmol/mol)	0.99 [0.97; 1.01]	0.473

Data are presented as OR [95% CI].

## Data Availability

The raw data are not available to the public according to rules of The Danish Data Protection Agency.
